# Germline Amino Acid Diversity in B Cell Receptors is a Good Predictor of Somatic Selection Pressures

**DOI:** 10.3389/fimmu.2013.00357

**Published:** 2013-11-08

**Authors:** Gregory W. Schwartz, Uri Hershberg

**Affiliations:** ^1^Systems Immunology Laboratory, School of Biomedical Engineering, Science, and Health Systems, Drexel University, Philadelphia, PA, USA; ^2^Department of Microbiology and Immunology, College of Medicine, Drexel University, Philadelphia, PA, USA

**Keywords:** B cells, somatic hypermutation, selection, diversity, evolution

## Abstract

The diversity of the immune repertoire is important for the adaptive immune system’s ability to detect pathogens. Much of this diversity is generated in two steps, first through the recombination of germline gene segments and second through hypermutation during an immune response. While both steps are to some extent based on the germline level repertoire of genes, the final structure and selection of specific receptors is at the somatic level. How germline diversity and selection relate to somatic diversity and selection has not been clear. To investigate how germline diversity relates to somatic diversity and selection, we considered the published repertoire of Ig heavy chain V genes taken from the blood of 12 individuals, post-vaccination against influenza, sequenced by 454 high-throughput sequencing. We here show that when we consider individual amino acid positions in the heavy chain V gene sequence, there exists a strong correlation between the diversity of the germline repertoire at a position and the number of B cell clones that change amino acids at that position. At the same time, we find that the diversity of amino acids used in the mutated positions is greater than in the germline, albeit still correlated to germline diversity. From these findings, we propose that while germline diversity and germline amino acid usage at a given position do not fully specify the amino acid mutant needed to promote survival of specific clones, germline diversity at a given position is a good indicator for the potential to survive after somatic mutation at that position. We would therefore suggest that germline diversity at each specific position is the better *a priori* model for the effects of somatic mutation and selection, than simply the division into complementarity determining and framework regions.

## 1 Introduction

The adaptive immune system’s ability to react to disease is based on the diversity of its immune repertoire. In the case of B cells, this diversity is generated in two rounds: the recombination of germline gene segments (V, D, and J for heavy chains, V and J for light chains) to create the B cell receptor (BCR), (1–3) as well as somatic hypermutation during an immune response (4–6). In both cases, these diversification processes are coupled with stringent somatic selection based on the binding affinity of the BCR (7). Thus, while the initial state of the BCR is at least somewhat based on an individual’s germline genes, the final structure of specific BCR mutants is based on somatic selection processes related to the binding affinity of the BCR. It remains unclear how selection and diversity at the germline level relate to selection at the somatic level. In this analysis, we demonstrate a link between the diversity and selection at the germline and somatic levels for V genes.

The germline genes encoding the different regions of the BCR are themselves diverse, even before considering the diversification produced from the recombination of different gene segments. Specifically in V genes, this diversity is non-uniformly spread across the gene sequence. Some positions always utilize the same amino acid in all V genes while others can utilize many different amino acids. This differential diversity is considered an indicator of the functional role of each position in the eventual tertiary structure of the receptor. Variable regions of the V gene sequences, called complementarity determining regions (CDR), are thought to be those that encode regions which interact with antigens, while the more invariant positions, called framework regions (FR), are proposed to be involved in the backbone of the receptor (8). It has been generally thought that somatic selection segregates along these two regions. Positive selection is thought to occur in the CDR, while mutations in the FR were mostly debilitating to affinity and lethal to the cell (5). It is now clear that this segregation is not strictly true – positively selected key mutations can be found in the FR (9) and negative selection can be seen in mutations throughout the sequence (10).

Previously, the diversity measurements of the receptors were based on differing diversity indexes with varying appropriateness and on partial sets of germline and mutant sequences (8, 11). We directly measured the “true” diversity of light chain V genes and heavy chain V genes (*V_H_*) based on the entire known BCR germline repertoire as found in the IMGT Ig gene database (12, 13). We demonstrated that the pattern of diversity in all V genes is non-uniform, with most positions showing a low level of diversity (2–5 relevant amino acids) and a few exhibiting higher levels of diversity (up to 10 relevant amino acids). If we rank all the positions in the sequence by their diversity, we can explicitly show that while the CDR is enriched for high diversity positions, many CDR positions have diversities as low as those found in FR and some FR positions have quite a high diversity (12). We previously suggested that it is the diversity of positions, not solely their association with the contiguous CDR or FR positions, which determines their functional role and the consequence of mutation.

The diversity of positions in the germline repertoire of V genes is the result of evolutionary selection of individuals and their progeny. The process of affinity maturation is based on somatic mutation and selection. It has thus far been unclear how these two processes of selection are related and if they can be connected at the V gene sequence level. To study this possible relationship, we considered a published dataset of ∼17,000 recombined BCR *V_H_* gene sequences from 12 individuals (14). We divided sequences by their clonotypes, identifying the clonal origin for each recombined sequence. In this way we could now count how many times each position was mutated in the repertoire. Comparing the number of individual times a position was mutated to its germline diversity (12), we found that while synonymous mutations were evenly spread across all positions, there was a clear positive correlation between the number of times a position had a mutated amino acid and that position’s diversity in the germline repertoire. From this we conclude that the diversity at the germline level is an indication for the potential for somatic harm as a result of mutation. The diversity of each specific position is a more direct measure of the functional consequence of mutation and selection at the somatic level than a mere division into CDR and FR.

## 2 Materials and Methods

### 2.1 Sequences analyzed

We analyzed the amino acid and nucleotide sequences of *Homo sapiens* BCR recombined *V_H_* genes (14). The sequences came from twelve healthy individuals post-vaccination against influenza (14). The individuals came from two age cohorts: 6 young (age range 19–45) and 6 old (age range 70–89). Sequences were acquired at days 0, 7, and 28 post-vaccination and included both IgG, IgM, and IgA class switched receptors. We divided the sequences into clones by fully aligning their nucleotide sequences to the germline V, D, and J genes from the IMGT Ig database (13). All sequences that shared the same germline source (V, J, and CDR3 length) were considered to be from the same clone. We filtered out sequences with ≥30 nt point mutations from the germline. This alignment resulted in the identification of 17,553 sequences divided into 9482 clones. Due to sequencing issues in the original dataset, we only analyzed the sequences from position 25 and on. IMGT numbering leaves gaps in order to remain consistent with all V genes. Also, the length of V genes is not always identical. Therefore, we only calculated germline diversity for amino acid positions 25 − 30, 35 − 59, 63 − 72, and 74 − 106, leaving us with 74 positions in the analysis. These positions were verified for adequate sampling by the use of rarefaction curves at each position (15). We considered a position viable if more than 99% of the curve consisted of a richness of ≥95% of the height of the curve (12). These curves rule out the possibility of having too many gaps in the germline repertoire.

We calculated the germline diversity per amino acid position using BCR *V_H_* genes collected from IMGT as in Ref. (12). We filtered out non-functional, partial, and duplicate sequences for the analysis. All sequences were numbered according to the IMGT unique numbering system based off of the universal alignment provided by IMGT (13). We defined CDR and FR positions as in Ref. (16).

### 2.2 Diversity measures

We measured the diversity of amino acids per position as in Ref. (12) with an order of diversity equal to 1. The process of measuring diversity is dependent on the order, or “Hill number” (17), we use during calculations. While measuring the effective number of species, the order affects the influence of the sample abundances. An order of 0 does not consider abundances, thus all types are considered equally (this is equivalent to the number of different types, also called “richness”). An order gives greater weight to rare species, while an order >1 gives greater weight to common species. When the order is 1, the effective diversity is determined without any bias (18). We previously described the result of analyzing the diversity of the different amino acid positions in the V gene germline repertoire at different orders of diversity (12). We decided here to focus on the order of 1 as we found no *a priori* reason to bias toward either the more commonly used amino acids at each position or toward the rare amino acids.

At each position *p*, the number of amino acids at that position was *N_p_* and the richness of the amino acids at that position was *R_p_*.

The measure of diversity used for these positions was “true” diversity *^q^D_p_*, where

(1) $${\;}^{q}D_{p}\equiv {\left(\overset{R_{p}}{\underset{i=1}{\sum }}{p_{i}}_{p}^{q}\right)}^{\left(1∕1-q\right)}$$

and *q* is the order of diversity and *p_i_* is the frequency of amino acid *i* (17, 18). At *q* = 1, equation (1) does not exist, however the limit as *q* approaches 1 is

(2) $${\;}^{1}D_{p}\equiv exp\left(-\overset{R_{p}}{\underset{i=1}{\sum }}{p_{i}}_{p}\ln{p_{i}}_{p}\right)$$

### 2.3 Definition of position categories

For every amino acid position, we counted – across all clones from any time point and person – the number of times a position changed amino acids and how many times that position maintained its amino acid from the germline. If a position was found to change into several amino acids in a single clone, that position was counted once for each different amino acid. The cases where amino acids were maintained relative to the germline were in some cases further divided into non-mutated and synonymous mutations. The amino acids collected in each category (changed, maintained, or synonymous mutation) were then further analyzed for their diversity and amino acid composition tendencies.

### 2.4 Correlations of diversity in germline positions versus changed or maintained amino acid position categories

Using a two sided Spearman’s rank correlation test, we assessed the correlation of germline diversity of human *V_H_* genes, as calculated in Ref. (12), with the counts and diversities of the three categories (changed amino acid, maintained amino acid, and synonymous mutation) described above.

### 2.5 Amino acid usage analysis

We assessed if position categories were biased toward specific amino acid usage types. Following our definitions of Ig relevant amino acid categorization by hydrophobicity and tendency to be found on the surface of the receptor (16, 19), we categorized amino acids as hydrophobic (IVLFCMW), neutral (AGTSYPH), and hydrophilic (NDQEKR) (12). We then categorized a position by how biased that position was to using amino acids from only one of these categories. If a position used only amino acids from one category, that position was considered to be of that type (i.e., a hydrophobic, a neutral, or a hydrophilic position). If the position had both neutral and one other category of amino acids, that position would be considered a “weak” version of that category (i.e., weak hydrophobic or weak hydrophilic). If there were amino acids in all categories, then that position was considered indeterminate. In all instances, if a position had a single amino acid in one category, and three or more in another category, the single amino acid category was ignored (12).

## 3 Results

### 3.1 Correlation of germline diversity to number of changed amino acids per position

**Figure 1:**
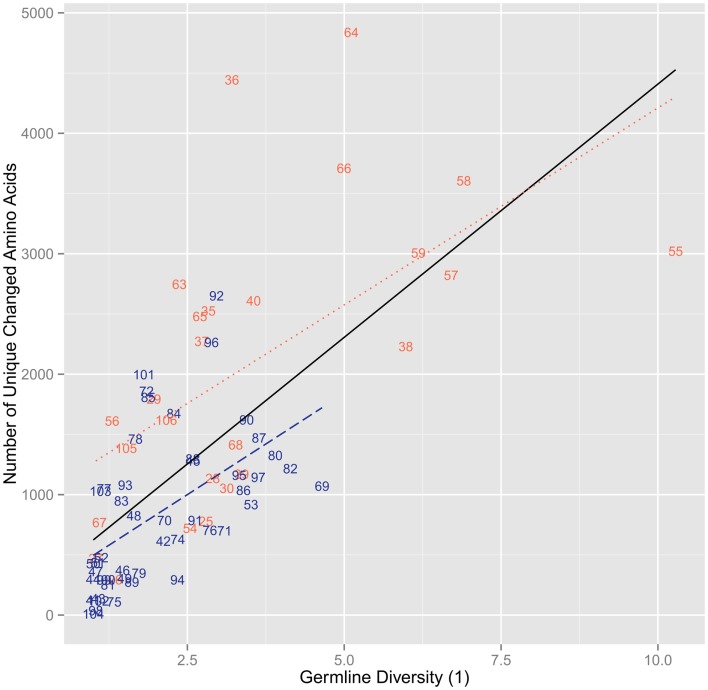
**The number of unique changed amino acids versus the diversity of order 1 of the germline sequences per position**. The points are labeled by their IMGT sequence position number and if they are found in the CDR (red) or the FR (blue). The lines represent linear regressions for all positions (black, *r*^2^ = 0.433), for FR positions (dashed blue, *r*^2^ = 0.289), and for CDR positions (dotted red, *r*^2^ = 0.349). We found a significant positive correlation for all positions (ρ = 0.710, *p* = 1.41 × 10^−12^). By correlating the positions based on FR and CDR, we found a significant positive correlation for both FR (ρ = 0.668, *p* = 2.17 × 10^−7^) and CDR (ρ = 0.676, *p* = 2.18 × 10^−4^).

When comparing the germline diversity at each position – as calculated from the prototypical IMGT database (12) – and the number of unique changed amino acids at each position, we find that these two properties are highly positively correlated (ρ = 0.710, *p* = 1.41 × 10^−12^). This correlation holds true if we consider CDR (ρ = 0.676, *p* = 2.18 × 10^−4^) and FR (ρ = 0.668, *p* = 2.17 × 10^−7^) positions separately and if we consider all positions as a whole (Figure 1). While this correlation is monotonic, it is by no means strictly linear as the linear model explains only ∼43% of variation in the extent of amino acid exchanges at the different positions (*r*^2^ = 0.433). Interestingly, while in general the CDR positions with similar diversity have more changed amino acids than most FR positions of similar germline diversity and the linear fits to CDR and FR are distinct, FR and CDR positions are not clearly separated in this plane (Figure 1).

**Figure 2:**
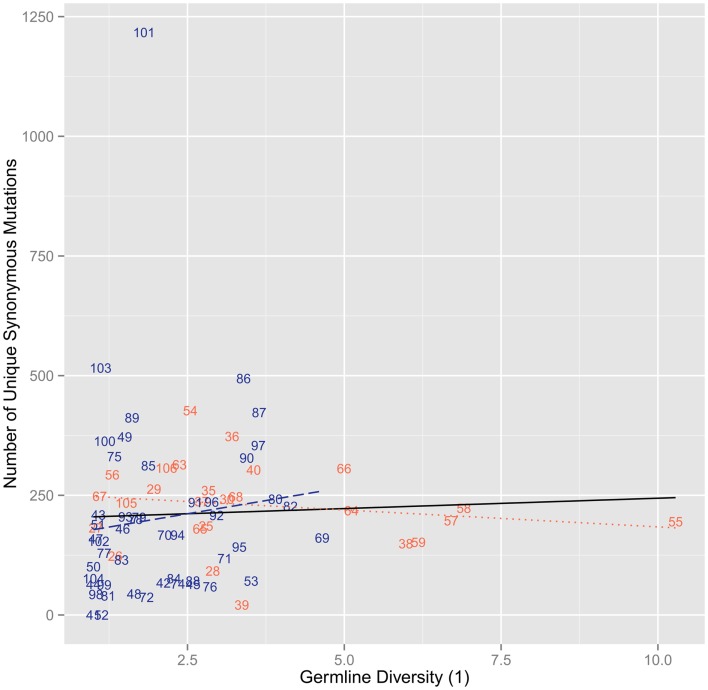
**The number of synonymous mutations versus the diversity of order 1 of the germline sequences per position**. We found no positive correlation with a flat trend (*r*^2^ = 1.87 × 10^−3^, ρ = 0.232, *p* = 0.0468). When splitting by region, we found no correlation for FR (*r*^2^ = 0.0127, ρ = 0.289, *p* = 0.0466) and CDR (*r*^2^ = 0.0322, ρ = − 0.223, *p* = 0.273).

The analysis with synonymous mutations shows no correlation (*r*^2^ = 1.87 × 10^−3^, ρ = 0.232, *p* = 0.0468) and similar mutation levels across the entire range of germline position diversities and no difference between CDR (*r*^2^ = 0.0322, ρ = − 0.223, *p* = 0.273) and FR (*r*^2^ = 0.0127, ρ = 0.289, *p* = 0.0466) positions (Figure 2). The results found using the diversity of the germline repertoire were at the whole repertoire level. No division into certain germlines was necessary and so the possibility for misidentification of the germlines by IMGT would have little to no impact on the diversity at the repertoire level. Moreover, when splitting up the analysis of clones by the germline they aligned with, there was no real difference in between different germlines and at the repertoire level (results now shown).

### 3.2 Correlation of germline diversity to changed or maintained diversity per position

**Figure 3:**
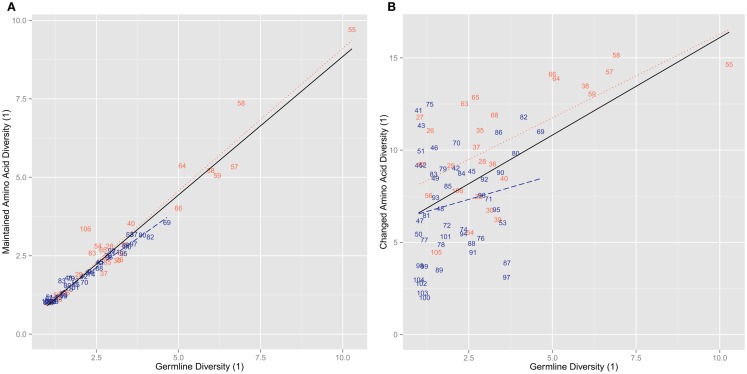
**The diversity of order 1 of the amino acids versus the diversity of order 1 for the germline sequences per position**. **(A)** The diversity of order 1 amino acid usage in amino acids positions. We found a significant positive correlation (*r*^2^ = 0.947, ρ = 0.961, *p* = 7.52 × 10^−42^). We also found positive correlations when the positions were split by region for FR (*r*^2^ = 0.970, ρ = 0.968, *p* = 3.67 × 10^−29^) and CDR (*r*^2^ = 0.937, ρ = 0.880, *p* = 1.76 × 10^−6^). **(B)** The diversity of order 1 amino acid usage of unique changed amino acids. We found a positive correlation (*r*^2^ = 0.284, ρ = 0.359, *p* = 1.70 × 10^−3^). When splitting the positions by regions, however, FR did not correlate with the germline diversities (*r*^2^ = 0.0347, ρ = 0.132, *p* = 0.371) while CDR did have a positive correlation (*r*^2^ = 0.419, ρ = 0.580, *p* = 2.27 × 10^−3^).

We next looked to see how the actual amino acid diversity of the mutant repertoire at the different positions related to the germline diversity. We found that the maintained positions had a diversity that was essentially identical to that found in the IMGT based germline repertoire (*r*^2^ = 0.947, ρ = 0.961, *p* = 7.52 × 10^4^2) (Figure 3A). In the changed positions a more complex pattern emerges. While overall we find again that there is a positive correlation between germline diversity and the diversity of the changed amino acids (*r*^2^ = 0.284, ρ = 0.359, *p* = 1.70 × 10^3^), the range of diversity is much greater in the changed positions (Figure 3B). This greater range of diversity is present in both CDR (*r*^2^ = 0.419, ρ = 0.580, *p* = 2.27 × 10^3^) and FR (*r*^2^ = 0.0347, ρ = 0.132, *p* = 0.371). However, when the FR is considered on its own this leads to a lack of significant correlation with germline diversity.

### 3.3 Changes in amino acid usage pattern

**Figure 4:**
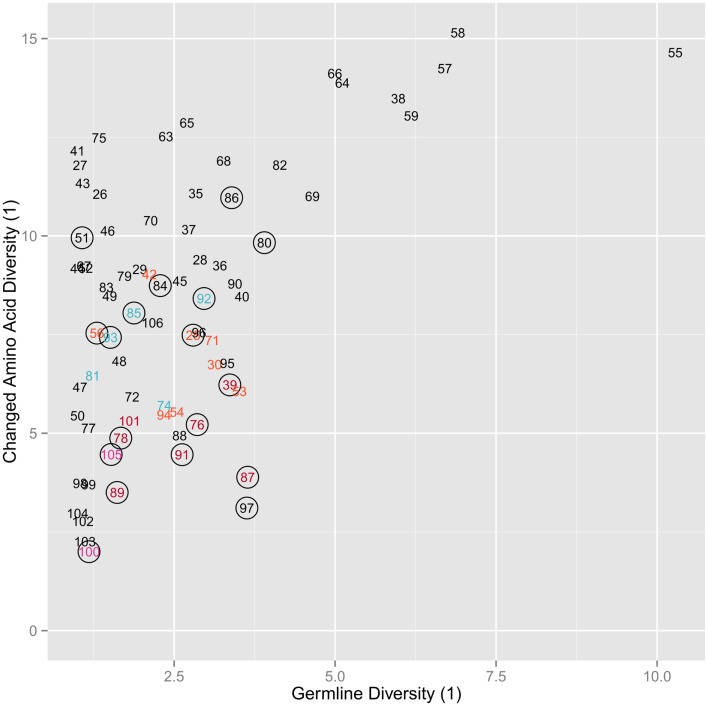
**Copy of Figure 3B annotated for amino acid usage bias**. The coloring for the labels signifies whether that position is hydrophobic (red), weak hydrophobic (light red), neutral (purple), weak hydrophilic (light blue), hydrophilic (blue), or indeterminate (black), while a circle around the position indicates that the position shares the same hydrophobicity bias as the equivalent germline position. All amino acids are changed.

We categorized the amino acid usage patterns for each position. We found in the maintained amino acid positions the biases toward using specific amino acid types are maintained. This was especially true for the positions in the germline that had stricter categories of amino acids usage bias. 13 out of 14 hydrophobic positions, 17 out of 19 neutral positions, and 8 out of 10 hydrophilic positions retain the same bias in the maintained positions as in the germline (Table 1). The positions with the more intermediate biases (weak hydrophobic and weak hydrophilic) in the germline did not adhere as strictly to the same bias category but tend to have changed to one of the neighboring biases. Weak hydrophobic becomes either hydrophobic or neutral. Weak hydrophilic becomes either hydrophilic or neutral (Table 1). Looking now at the changed position we see that biases change much more (Figure 4). Most positions simply become indeterminate (i.e., have no clear bias). However, it is interesting to note that those positions that do have some bias exhibit either exactly the same bias as they have in the germline repertoire or one that is similar (Table 1).

**Table 1:** **Number of positions for each amino acid usage bias**.

Category	Germline	Maintained							Changed						
Hydrophobic	14	16:	(13,	3,	0,	0,	0,	0)	7:	(6,	1,	0,	0,	0,	0)
Weak hydrophobic	12	0:	(0,	0,	0,	0,	0,	0)	8:	(5,	2,	1,	0,	0,	0)
Neutral	19	27:	(0,	6,	17,	4,	0,	0)	2:	(0,	0,	2,	0,	0,	0)
Weak hydrophilic	13	3:	(0,	0,	1,	2,	0,	0)	5:	(0,	0,	1,	3,	1,	0)
Hydrophilic	11	10:	(0,	0,	0,	1,	8,	1)	0:	(0,	0,	0,	0,	0,	0)
Indeterminate	5	18:	(1,	3,	1,	6,	3,	4)	52:	(3,	9,	15,	10,	10,	5)

The numbers in parentheses after the colon signify the classifications of the respective positions in the germline repertoire: hydrophobic, weak hydrophobic, neutral, weak hydrophilic, hydrophilic, indeterminate.

## 4 Discussion

The specificity of B cell and T cell receptors, while based on genes in the germline, is ultimately not of the germline template. Due to the imprecise nature of V(D)J recombination and, in B cells, somatic mutation, the final affinity of each immune receptor is neither inherited nor heritable. For this reason it is difficult to assess how germline diversity and its selection relate to selection during an immune response and specifically how they relate to the anticipated outcome of somatic mutation during an immune response. We have previously shown that the diversity of the germline V gene repertoire can be characterized by looking at the amino acid diversity of individual positions in the V gene sequence (12). The distribution of diversity is non-uniform with most, but not all highly diverse positions being found in the CDR. Furthermore, different positions show different biases toward the use of hydrophobic or hydrophilic amino acids (12). To contrast this picture of germline diversity with somatic changes, we have taken a published sample of the human peripheral B cell repertoire following influenza vaccination. We divided all of the sequences in this dataset into their respective clones and counted the number of times each position in the V gene sequence changed or maintained the amino acid found in the germline origin of its clone. By doing so, we could compare for each position how it contributes to repertoire diversity and its selection when changed from its germline and when it remained the same. Analyzing the maintained positions and their diversity allows us to ask to what extent clonal shift changes the diversity of the repertoire from the germline while analyzing the changed positions describes the effects of selection.

Starting with the maintained positions, we see that the germline diversity exhibited in the prototypic repertoire in the IMGT database, which does not assume any specific biases in *V_H_* usage, is recapitulated in even the small and clonally shifted snapshot of the immune repertoire analyzed here. We find a significant linear correlation between germline diversity and that of positions with maintained amino acids (Figure 3A) and also clear conservation of amino acid usage biases (Table 1). Thus, despite the fact that we analyzed ∼1000 clones per person (out of potentially 10^11^ clones) with a significant shift toward certain *V_H_* genes, we still identify more or less exactly the same diversity and amino acid usage as described by the IMGT database. This suggests to us that clonal shift does not change the make up of amino acid diversity in the B cell repertoire. Furthermore, the existing IMGT database of human V genes represents this positional diversity well.

With regard to the changed positions, we find that there is a significant positive correlation between the level of diversity in the germline at a specific position and the survival of clones with changed amino acids at that position (Figure 1). Such a correlation suggests that there is a relationship between the tendency to diversify a position at the germline level over evolutionary time and the likelihood of mutants at those positions to survive somatic mutation and selection. We do not find any kind of correlation between germline diversity and synonymous mutation level (Figure 2). For this reason, while the exact observed levels of mutations and surviving mutants with specific amino acid changes may have also been influenced by biases in somatic mutation targeting or sequencing error, these explanations could not be the only reasons for our results. It would be unreasonable to think that mutation bias and sequencing error would only influence non-synonymous mutation rates and so it is thus quite clear that selection is causing this skew in mutant numbers. While assessing the exact rate of selection is beyond the scope of this paper, we can attempt to use these levels of synonymous mutations to estimate some ballpark level of expected non-synonymous mutations, which under neutral conditions we would assume to be three times as high. We can then see that all the positions with lowest germline diversity must be undergoing quite stringent negative selection and that once germline diversity gets higher (>2) there are some positions that appear to also be undergoing some positive selection. The positions with a germline diversity value >5 show rates of non-synonymous mutations 10- to 20-fold greater than synonymous mutations – a clear indicator of strong positive selection.

Another indication that specific positive selection has great influence on the final level of amino acid changed at each position is that the diversity of changed positions is much higher than the germline diversity at those positions (Figure 3B). Furthermore, in most cases their bias in usage is indeterminate (Figure 4). Thus, while the likelihood of survival is related to germline diversity, the specific change in amino acid that is needed to save the clone is also determined by the specific selection interactions in which that change was positively selected. However, it is worth noting that positions that can be classified (i.e., are not indeterminate) in the mutants all exhibit the same general amino acid bias as the germline repertoire (Table 1).

Taking all of these findings into account, we propose that germline diversity is a good indicator of the likelihood of survival following mutation but cannot account for the specific amino acid whose usage accounted for survival of a specific clone, although this usage can be approximated. This usage is based on the specific affinity maturation event and immune response that leads to the formation of the clone. We would further conclude that while CDR and FR do roughly segregate the sequence, a better measure of potential selection force is the specific germline diversity of each position. This is especially true for positions with <5 diversity in their germline amino acids. In such positions, while diversity indicates a range of possible levels of surviving mutants, there is no clear distinction between positions in the CDR and the FR. Indeed, the only reason one exists beyond diversity of 5 is that no FR positions have such high germline diversities.

## Conflict of Interest Statement

The authors declare that the research was conducted in the absence of any commercial or financial relationships that could be construed as a potential conflict of interest.
